# Adaptive Hyperactivity and Biomarker Exploration: Insights from Elders in the Blue Zone of Sardinia

**DOI:** 10.3390/jcm13216451

**Published:** 2024-10-28

**Authors:** Alessandra Scano, Germano Orrù, Goce Kalcev, Massimo Tusconi, Maura Spada, Laura Atzori, Caterina Ferreli, Flavio Cabitza, Diego Primavera, Federica Sancassiani

**Affiliations:** 1 Department of Surgical Sciences, Oral Biotechnology Laboratory (OBL), 09042 Cagliari, Italy; alessandrascano@libero.it; 2 Azienda Ospedaliero-Universitaria di Cagliari (AOU Cagliari), 09042 Cagliari, Italy; massimotusconi@yahoo.com; 3 Department of Medical Sciences and Public Health, University of Cagliari, 09042 Cagliari, Italy; gocekalcev@yahoo.com (G.K.); m.spada12@studenti.unica.it (M.S.); diego.primavera@unica.it (D.P.); federicasancassiani@yahoo.it (F.S.); 4 The National Alliance for Neuromuscular Diseases and Neuroscience GANGLION Skopje, 1000 Skopje, North Macedonia; 5 Dermatology Clinic, Department of Medical Sciences and Public Health, University of Cagliari, 09042 Cagliari, Italy; atzoril@unica.it (L.A.); ferreli@unica.it (C.F.); 6 Fondazione per la Tutela dell’Identità Ogliastrina, Corso Vittorio Emanuele II, Perdasdefogu, 08046 Nuoro, Italy; cabitza.flavio@gmail.com

**Keywords:** bipolar disorders, BD, laboratory medicine, applied biomedical technologies, bipolar spectrum, prevention, new technologies, hyperactive, blue zone, biomarker

## Abstract

**Background/Objectives:** Adaptive hyperactivity characterized by increased activity levels and novelty-seeking traits without mood disorders is prevalent among older adults in Sardinia’s “blue zone,” an area with high longevity. This study aims to evaluate the adaptive nature of hyperactivity concerning quality of life, social rhythms, and mood symptoms in individuals from this region, particularly among elderly adults over 80. **Methods:** This observational cross-sectional study included adults and older adults over 80 from Sardinia’s blue zone. This study included a sample of patients followed at the Center for Consultation Psychiatry and Psychosomatics for Bipolar Disorder of the University Hospital of Cagliari and a homogeneous comparison sample of patients without psychiatric pathologies, referred to the Dermatology Clinic of the same hospital, for a period of 6 months, from February to August 2024. The general sample, divided into two parts—cases, represented by patients with psychiatric pathology, and controls, patients without psychiatric pathology—was divided in turn into three sub-groups: “adults” (18–64 years), young elders (65–79), and old elders (over 80 years). The participants underwent psychiatric interviews and completed the Mood Disorder Questionnaire (MDQ), Patient Health Questionnaire (PHQ-9), SF-12, and Brief Social Rhythm Scale (BSRS). Data were compared with national and regional normative data. **Results:** Older adults in the blue zone demonstrated higher MDQ positivity (22.58%) compared to the national averages (0.87%), without corresponding increases in dysregulated rhythms, depressive symptoms, or reduced quality of life. Younger old persons (65–79 years) showed increased rhythm dysregulation (BSRS score: 20.64 ± 7.02) compared to adults (17.40 ± 6.09, *p* = 0.040), but this trend was not observed in the oldest group (80+ years). No significant differences were found in the CH3SH and (CH3)2S levels between groups. **Conclusions:** The hyperactivity observed in older adults from Sardinia’s blue zone appears adaptive and not linked to social rhythm dysregulation, depressive symptoms, or a diminished quality of life, suggesting resilience factors which may contribute to longevity. These findings support the potential classification of such hyperactivity as beneficial rather than pathological, warranting further research into biomarkers and psychoeducational interventions to prevent the onset of bipolar disorders in predisposed individuals.

## 1. Introduction

Research has shown that older adults with hyperactivity and novelty-seeking traits, without mood disorders and with a good level of social inclusion, show some genetic characteristics found commonly in bipolar disorders [1,2]. Starting from this evidence, attempts have been made to classify hyperactivity on a continuum from adaptive hyperactivity useful for overcoming challenging conditions [3] to hyperactivity with stress and the dysregulation of rhythms but without a precise psychiatric diagnosis (“DYMERS syndrome”), and, finally, pathological hyperactivity in the context of bipolar mania [4].

Verification has also been carried out using the Mood Disorder Questionnaire, a tool created for the screening of bipolar disorder but which, according to current classifications [5,6,7], is not considered very accurate in recognizing cases diagnosed as bipolar disorder. In this case, individuals with “non-pathological” hyperactivity but with traits of exploration and search for novelty were identified among the so-called “false positives” (i.e., positives without bipolar disorders) [8], similar to the case of migrants in Latin American megacities compared to the resident population in rural Europe [9,10], or people without a diagnosis of bipolar disorder but with the dysregulation of rhythms, a compromised quality of life, or DYMERS syndrome [11]. Thus, positives on the MDQ ranged from people with adaptive hyperactivity (i.e., hyperactivity well aimed at their own goals of life, with which they were satisfied) to people under stress. However, without a clear psychiatric diagnosis, stress manifested itself with the dysregulation of social rhythms, symptoms of sub-threshold anxiety–depression, and poor life satisfaction in people with genuine bipolar disorder. In this study, the MDQ is used to identify those who present traits of non-pathological hyperactivity among people without bipolar disorder [4].

The phenomenon of adaptive hyperactivity in aging populations, particularly in long-living populations like the elders of Sardinia’s blue zone ([12,13,14]), represents a unique area of study, with significant implications for understanding longevity and mental health resilience. Research into potential biological markers associated with hyperactivity in these populations could reveal underlying mechanisms that contribute to their remarkable life expectancy and quality of life [15,16,17,18,19]. Despite the growing body of literature on longevity, few studies have explored the connection between adaptive hyperactivity and clinical biomarkers in elderly populations. This gap underscores the importance of examining whether specific biological and psychological markers—such as volatile sulfur compounds, mood dysregulation scales, and rhythm assessment tools—might correlate with or explain these adaptive traits.

Our study aims to address this research gap by integrating assessments through multiple validated tools. In addition to the Mood Disorder Questionnaire (MDQ), which is employed to measure hyperactivity traits potentially associated with the bipolar spectrum, we also use the Patient Health Questionnaire (PHQ-9) and the Short Form-12 Health Survey (SF-12). The PHQ-9 allows us to evaluate depressive symptoms, while the SF-12 provides a measure of perceived quality of life, thereby broadening our understanding of mental health in this demographic [20,21,22,23,24]. The inclusion of these tools provides a comprehensive evaluation of both mood symptoms and overall health, reducing the focus solely on the MDQ and allowing for a more nuanced assessment of adaptive hyperactivity [14].

Investigating these traits among Sardinia’s elders may also offer insights into the adaptive biological and psychological factors that contribute to resilience against mood disorders. Understanding these adaptive mechanisms is essential, as it may inform interventions aimed at promoting mental well-being and quality of life in aging populations elsewhere. By connecting clinical and biological markers to behavioral traits like hyperactivity, our research seeks to enhance knowledge about protective factors that support longevity and mental health resilience, thus bridging an important gap in current geriatric psychiatry and epidemiology literature.

This line of research began with hyperactive older adults. This was perhaps inevitable since our group works in one of the so-called “blue zones”. These are the five areas of the world where life expectancy is significantly higher than the world average. A study by Poulin et al. [12] found that central Sardinia is the area with the highest concentration of centenarians in the world. During the aforementioned investigation, the researchers drew blue circles on a world map to identify the areas with the highest longevity, hence the term “blue zone”. In addition to Sardinia, the “blue zones” identified were the following: the island of Okinawa, Japan; Loma Linda, California, U.S.A.; the Nicoya Peninsula in Costa Rica; and, finally, the island of Icaria, Greece [25]. This is why, for a group dealing with hyperactivity in Sardinia, it was inevitable that the study would involve older adults. One of the causes of longevity in our blue zone is considered to be maintaining a high level of activity, even in leisure activities, despite the advancing years [26]. This study was geared toward the possibility of assessing whether the hyperactivity observed in older adults in the blue zone of Sardinia appears adaptive and not related to social rhythm dysregulation, depressive symptoms, or a decreased quality of life. Such observations would allow the highlighting of possible resilience factors that may contribute to longevity. The present work aims to verify the state of hyperactivity concerning the evaluation of the quality of life, social rhythms, and mood symptoms (therefore evaluating the state of adaptivity) in a sample of people from the Sardinian blue zone, with a strong representation of older adults and people over eighty, recruited as a control group for clinical research.

## 2. Methods

### 2.1. Design

Observational Cross-Sectional Study.

### 2.2. Sample

The sample consisted of controls recruited for a clinical study [27] of people without a diagnosis of bipolar disorder according to the *DSM-5* [28] in order to verify the state of hyperactivity and well-being with particular attention to the elderly over 80 years of age. The study included a sample of patients followed at the Center for Consultation Psychiatry and Psychosomatics for Bipolar Disorder, and a homogeneous comparison sample of patients without psychiatric pathology, referred to the Dermatology Clinic of the same hospital, for a period of 6 months, from February to August 2024. The general sample, divided into two parts—cases, represented by patients with psychiatric pathology, and controls, patients without psychiatric pathology—was divided in turn into three sub-groups: “adults” (18–64 years), young elders (65–79), and old elders (over 80 years) [29,30]. After a detailed explanation of the objectives of the survey and the signing of the informed consent form, the people who agreed to participate underwent a psychiatric interview and a general anamnesis and filled out the study instruments. The inclusion criteria for the control sample were an age over 18 years without any exclusion by gender. In addition, for the specific interests of this study, we recruited people over 80 years of age from the central area of Sardinia (blue zone) on the same days and in the same outpatient dermatological facility.

All psychometric tests, including the MDQ+, PHQ-9, SF-12, and BSRS, were administered by trained and specialized personnel, ensuring consistency in application and adherence to standardized procedures. Prior to participation, each subject provided informed consent, acknowledging an understanding of the study’s purpose and procedures. The administration process was designed to minimize variability, with personnel following strict protocols for each tool. For example, the MDQ+ was utilized to assess symptoms aligned with the bipolar spectrum, while the PHQ-9 evaluated depressive symptoms. The SF-12 provided insights into health-related quality of life, and the BSRS measured social rhythm regularity. Each instrument’s results were interpreted according to established scoring criteria, which facilitated objective and consistent data analysis. This rigorous approach aimed to reduce any impact of variability on the study’s findings, ensuring that the results would reflect true associations rather than methodological inconsistencies.

#### 2.2.1. Study Tools

##### Mood Disorder Questionnaire (MDQ)

The MDQ has been useful for measuring the level of hyperactivity. The MDQ was initially created as a screener for bipolar disorder. However, the instrument’s accuracy has been shown to be poor, with the screening of an excess of false positives [5,6]. However, as mentioned above, it has recently been discovered that the so-called false positives were people with traits of hyperactivity that ranged from people with adaptive hyperactivity (i.e., with hyperactivity well aimed at their own goals of life, with which they were satisfied) to people under stress. However, without a clear psychiatric diagnosis (references [31,32,33]), stress manifests itself with the dysregulation of social rhythms, symptoms of sub-threshold anxiety–depression, and poor life satisfaction in people with genuine bipolar disorder [7]. In this study, the MDQ was used to identify people presenting traits of non-pathological hyperactivity among those without bipolar disorder [4].

##### Patient Health Questionnaire (PHQ9)

The PHQ9 includes items about behavior, mood, attitudes, and thoughts typical of bipolar disorder. A threshold for determining whether the proband has characteristics of the bipolar spectrum is to score positively in at least seven of the thirteen items on the questionnaire [4,34].

The measure of depressive symptoms (sub-threshold, as sample selection excluded those who had a lifetime psychiatric diagnosis) was the score obtained on the “Items” version of the Patient Health Questionnaire (PHQ9) [35,36] in its Italian version [37]. The overall score of the PHQ9 scale is the sum of the scores of each of the nine items of the tool. Each item inquires about one of the core symptoms useful for making a diagnosis of depressive episodes according to the DSM-5 (Diagnostic and Statistical Manual of Mental Disorders) [28]. Kroenke [35] found that a score higher than 9 identified people with clinically relevant features of depression. Due to the characteristics of our sample and its aims, we only compared the means of the scores between groups.

##### Short-Form Survey (SF-12)

The SF-12 is a measure of the perceived health-related quality of life. The instrument includes questions covering physical and mental health domains, investigating aspects of physical health and functioning, limitations in life due to physical and emotional health, presence of bodily pain, perception of general health, social functioning, vitality, and mental health [38]. The SF-12 has been used in various populations and countries in studies on health and disease [39]. For this study, we used the comparison between the total SF-12 scores and the presence in sub-groups of the individuals below the mean and one standard deviation score of a representative sample of the Italian population [40,41].

##### Brief Social Rhythm Scale (BSRS)

The Brief Social Rhythm Scale (BSRS) [42] is a shortened and simplified tool derived from the Social Rhythm Metric (SRM) [43]. The BSRS aims to assess the (ir)regularity of activities in daily life (sleeping, eating, and having social contacts at work if people have a job and/or during leisure time). The tool consists of ten questions inquiring about the previous week’s behavior: each item/activity is coded on a scale from 1 (maximum regularity) to 6 (maximum irregularity). Previous studies found that the BSRS had excellent internal consistency in different languages. We adopted the Italian-validated version of the BSRS [44].

##### Analysis of Volatile Sulfur Compounds (VSCs)

The breath of all subjects involved in this study was analyzed for three distinct sulfur volatile compounds (VSCs). The sampling was performed by inserting a sterile syringe up to the stopper into the oral cavity and holding the syringe between the front teeth; after the patient’s lips had been closed for 30 s, 1 mL of oral air was extracted. Following this extraction, we promptly inserted the sample into the inlet of a portable gas chromatograph apparatus (Oral Chroma, ABI Medical, Abilit Corp., Osaka, Japan). After about eight minutes the amounts of (i) hydrogen sulfide (H_2_S), methyl mercaptan CH_3_SH, and dimethyl sulfide (CH_3_)_2_S were displayed by the apparatus. An excessive amount of these metabolites suggests a dysbiosis condition in the oral and gastric tissues. For example, oral dysbiosis was found to be more prevalent amongst subjects without regular habits, particularly in patients with alimentary disorders or psychotic drug assumption, a condition often related to mood disorders [45].

### 2.3. Statistical Analysis

Data were collected anonymously using I.D. identification numbers. The ANOVA one-way measure was used to measure differences in the mean and standard deviation scores of numerical data, and the Chi-square test (with Yates correction if needed) or Fisher’s exact test were used to compare the nominal data.

Before performing the ANOVA, we verified the assumptions required for this analysis, including the normality of the distribution and the homogeneity of variances. Normality was assessed using the Shapiro–Wilk test, while Levene’s test was employed to evaluate the equality of variances across groups. Where assumptions were not met, we applied non-parametric alternatives or appropriate corrections to ensure the validity of the results.

The scores on the MDQ, SF-12, and PHQ9 of the study sample (subdivided into adults and elders) were compared with the mean and standard deviation of similar surveys concerning national M.D.Q. [8] and SF12 [41] or regional samples [46].

Then, the scores of each sub-sample group (subdivided into “adults”, “young elders”, and “old elders”) were compared using all study tools. Comparison of the frequency means scores for PHQ9, SF-12, and MDQ positivity in our sample with data from the general population could only be conducted for the group of elderly people over 64 years of age because frequency among old people is not reported in the results of the Italian study.

While comparing means provides a general overview of differences between groups, this method has inherent limitations. It does not account for variability within groups, which may obscure individual experiences and lead to an incomplete understanding of the data. For example, two groups with the same mean score can exhibit substantially different score distributions, with one group displaying a narrow range and the other a broad range. Such differences in variability are important, as they may indicate distinct patterns or sub-groups within the data that are not visible through mean comparisons alone. Similarly, a higher mean score in one group does not necessarily represent the experiences of all individuals within that group; individual scores may vary widely around the mean, suggesting diverse experiences and responses.

To address this limitation, future analyses could include measures of dispersion, such as standard deviation and interquartile range, to better capture the variability within each group. Additionally, assessing data distribution characteristics, such as skewness and kurtosis, would provide a fuller picture of the range of experiences captured by each psychometric tool. These considerations underscore the importance of complementing mean comparisons with a broader set of statistical descriptors to ensure a nuanced interpretation of results.

All statistical analyses were conducted using the Stata software (version 17.0; StataCorp LP, College Station, TX, USA). Chi-squared tests with Yates’ correction for continuity were applied for categorical data to assess associations between variables, while continuous variables were analyzed using one-way ANOVA, with Fisher’s correction employed when necessary. This approach ensured robust comparisons across groups and adjusted for potential biases in smaller sample sizes.

### 2.4. Ethical Aspects

Institutional Review Board Statement (IRB) approval was not required for this study because the data were de-identified and made available to the public. This study was conducted according to the guidelines of the 1964 Helsinki Declaration. All the study participants signed a written informed consent form after receiving a detailed description of the study (aims, procedures, and data protection), and they were aware that the study could be terminated at any time.

## 3. Results

Table 1 illustrates the characteristics of the study sample divided into the sub-groups of “adults” (18–64 years), young elders (65–79), and old elders, the latter all from the blue zone (Central Sardinia). The three sub-samples did not present significant differences in distribution by sex despite a slightly lower presence of women in the sample of old elders, which, however, did not reach statistical significance. In the elderly population, the frequency of positives in the MDQ results (in the absence of psychiatric diagnoses) was significantly higher than the Italian normative average [22.58% vs. 0.87%, Chi-square, 1df = 155.83, *p* < 0.0001].

Table 2, Table 3 and Table 4 compare the mean and standard deviation in the answers of the study sample variables (subdivided into adults and elders [>64 years old]) with available national (MDQ and SF-12) or regional (PHQ9) normative data. The adult sample of the present study shows a frequency of depressive symptoms (PHQ9) and positive results on the MDQ, which are homogeneous with the normative reference samples. The frequency of the SF12 scores is higher than the reference Italian population (36.14 ± 5.28 vs. 38.61 ± 6.33, one-way ANOVA 1;653 d, with Bonferroni correction, F = 4.954, *p* = 0.026). The elderly in our sample are, instead, homogeneous with the normative samples for the MDQ and PHQ9 scores. In our study sample, no differences appear between the elderly and adults regarding the PHQ9 score, which is in line with what happens in the normative sample. The Italian normative sample shows a higher score for adults on the SF-12 compared to the elderly (38.61 ± 6.33 vs. 34.32 ± 7.20, one-way ANOVA, with Bonferroni correction, 1;2000 df; F = 144.7; *p* < 0.0001), but a similar difference does not emerge in our study sample, where the SF-12 scores are homogeneous in the two age groups. In the normative sample, the comparison of positive MDQ results by age shows a lower frequency in the elderly compared to younger adults (0.87% vs. 3.57%, OR = 0.24 CL 95% 0.1–0.5), but, in our sample, the ratio is inverted (22.58% vs. 6.06%, OR = 4.52 CL 95% 1.0–21.3).

Table 5 compares oral biomarkers (VSCs), well-being and/or impairment, and mood suffering within the sub-groups of the study sample. Once the sample of elderly people was divided into young elderly and older elderly people, it was noted that the positivity on the MDQ increased in young adults compared to older adults (but the difference did not reach statistical significance), the frequency was even higher in old elderly people, and, in this case, the difference compared to adults was statistically significant (28.57% vs. 6.45%, Fisher exact tests, *p* = 0.031). In a specular way, the BSRS score was higher in young elderly people than in adults, indicating a dysregulation of rhythms (20.64 ± 7.02 vs. 17.40 ± 6.09, one-way ANOVA, 1;72 df with Bonferroni correction F = 4.376, *p* = 0.040), but the same score decreased in old elderly people, and no difference was detected with adults. A similar trend emerged from the frequency of people with an SF-12 score lower than the mean minus one standard deviation of the Italian normative value [40]; in this case, too, the frequency was higher among young-old people than among adults (31.70% vs. 12.1%, Fisher exact test, *p* = 0.041). However, it decreased again with the old groups. In this case, any statistical significance was eliminated when comparing the adult scores. The distribution of the PHQ9 scores and volatile sulfur compounds in the expired air did not show any statistically significant differences between groups; however, the distribution of (CH_3_)_2_S showed a distribution profile between groups similar to that of the BSRS, with an increase in the young elderly and a decrease in old blue-zone elderly.

## 4. Discussion

Our study shows that a sample of old elders from Sardinia’s blue zone, the area with the highest density of centenarians in the world, have levels of hyperactivity, measured with the MDQ, which are paradoxically higher than those of the adults of the comparison sample. in which the general trend showed a lowering of the score as age increased [40,41,47]. However, this high score and the increasing prevalence of positives did not correspond to the worsening in the dysregulation of rhythms [48,49], the increase in the score on the PHQ9 (depressive symptoms) [50], and volatile sulfides in breath [27], nor is there an impairment in the perception of quality of life and well-being as one would have expected in the case of an increase in the frequency of bipolar disorders [40,41,51].

These findings suggest that the hyperactivity in this elderly population is likely adaptive and not pathological, warranting further exploration into its potential role in the longevity and mental health resilience seen in this unique demographic.

Adjustments for multiple comparisons, such as corrections for family-wise error rates, were not implemented in this study. This omission is recognized as a limitation, as it may impact the reliability of certain results by increasing the likelihood of type I errors. Additionally, effect sizes and confidence intervals were not included in the current analysis, which limits the ability to interpret the magnitude and precision of the observed effects. We recognize the importance of these metrics for providing a more nuanced understanding of our findings and plan to incorporate them in future studies to enhance the robustness and clinical interpretability of the results. These enhancements will improve the rigor and comprehensiveness of subsequent analyses.

The result of our study, therefore, supports the conclusion that the hyperactivity identified in the old elderly from Sardinia’s blue zone with a positive result on the MDQ can be classified as adaptive. It is not associated with the dysregulation of social rhythms as in the case of a positive result on the MDQ in the so-called syndrome of dysregulation of social rhythms and disability [4,52] nor with an increase in “sub-threshold” depressive symptoms or a lowering of the perception of quality of life as often occurs in mania and mixed bipolar states also associated with MDQ positivity [29,53].

This study has several limitations that should be acknowledged, as they may impact the interpretation and generalizability of the findings. First, the relatively small sample size, particularly in the “old old” group (*n* = 21), limits the statistical power of the study and restricts the ability to generalize the results to a broader elderly population. While our findings provide valuable insights into the association between adaptive hyperactivity and quality of life in Sardinia’s oldest residents, a larger sample would be necessary to confirm these observations and extend them to other populations.

Another limitation is the lack of control for confounding factors that could influence hyperactivity and mental health outcomes, such as medication use, co-existing medical conditions, and lifestyle differences. Medication, in particular, can significantly affect mood and activity levels, as can chronic health conditions and variations in physical activity patterns. Additionally, the influence of psychological stressors was not directly assessed; yet, chronic stress is a known factor in mood dysregulation and may alter hyperactivity levels in aging populations [54,55,56,57,58]. Future studies should consider controlling for these variables to better isolate the role of adaptive hyperactivity in promoting longevity.

Lastly, cultural attitudes towards mental health and aging in Sardinia may influence how symptoms are reported, potentially introducing a bias in symptom expression. Sardinia is renowned for its high density of long-lived individuals, which may shape local perceptions of aging and mental health in unique ways. This cultural context might lead individuals to view certain symptoms, such as increased energy or mood variability, as normative or even beneficial, rather than as markers of pathology. Consequently, the results may reflect a cultural adaptation rather than a universal phenomenon, underscoring the need for comparative studies in diverse cultural contexts to validate these findings [59,60,61,62,63].

In addition, the MDQ+ results for the “young old” group did not reach statistical significance after correction (*p* = 0.088). However, this result, while not strictly significant, shows a trend toward statistical significance, which may be clinically meaningful and warrants further consideration. This suggests that this study may lack sufficient power to detect true differences in hyperactivity levels within this sub-group. The relatively small sample size and data variability could have limited the ability to capture subtle associations. Future studies with larger sample sizes would enhance statistical power, providing a more reliable assessment of differences between age groups and further clarifying the role of adaptive hyperactivity across varying stages of aging.

The frequency of MDQ positivity in our sample compared with the data of the general Italian population only considered the group of elderly people over 64 years of age, since frequency among old older people is not reported in the results of the Italian study. Although this “dilutes” the frequency of our sample of old older people within the entire sample of elderly people, the result of the comparison is still exceptional; instead of a reduction of 1/4 in the risk of MDQ positivity as found in the national sample, the elderly people in our sample showed a risk that was over four times higher, and frequency was observed even if the comparison with the markers clarified that the highlighted condition was not at all pathological.

The high frequency of MDQ+ positivity among Sardinian elders likely reflects a unique combination of cultural, environmental, and genetic factors specific to Sardinia’s blue zone, an area recognized for its exceptional longevity [58,64,65,66,67]. Sardinia’s blue zone is characterized by a distinct lifestyle and close-knit community structure, which may promote social cohesion and resilience, potentially influencing mental health outcomes in ways which differ from those in other populations. Additionally, the genetic makeup of this population may contribute to the observed traits, as previous research suggests that certain genotypes associated with mood and activity levels are more prevalent in long-living populations.

Given these specific influences, generalizing our findings to broader or culturally distinct populations could be misleading. It is essential to contextualize these results within the unique environment and lifestyle of Sardinia’s elders. Future studies conducted in diverse populations and environmental contexts will be necessary to determine whether the patterns observed here are indeed specific to this blue zone or indicative of broader adaptive mechanisms linked to longevity and mental health resilience.

This model suggests that individuals with these traits, even in the absence of full-blown mood disorders, may represent subclinical manifestations or early expressions of a bipolar condition. At the lower end of this spectrum are those with adaptive hyperactivity and enhanced activity levels, traits which can become maladaptive under stress or with disrupted social rhythms.

The result, therefore, indirectly confirms the theory which suggests that MDQ positivity defines an area linked to the historical neo-Kraepelinian concept of the bipolar spectrum [68,69], which includes people with adaptive hyperactivity, syndromes of the dysregulation of rhythms linked to chronic stress, and cases of genuine bipolar pathology [4,52]. These findings align with the idea that the bipolar spectrum includes not only individuals with clear bipolar disorder but also those displaying sub-threshold traits or adaptive behaviors underpinned by hyperactivity.

Despite the limitations of the small sample, selected from patients who had to undergo a routine dermatological visit, this study lays the foundation for future research studies on biomarkers of adaptive hyperactivity. This may have important consequences in the geriatric and psychiatric fields in the prevention of bipolar disorder. The study of how hyperactive elderly people from the blue zone have addressed their hyperactive attitudes could, in fact, suggest important elements for developing psychoeducational training for the prevention of the onset of bipolar disorder in individuals with these “basic” characteristics [70].

A first element that must be underlined is the concomitance between hyperactivity (positive on the MDQ) and the good regulation of rhythms (such as sleep, eating, and meeting friends), which seems, in fact, to be the key element. Indeed, both in the recently described DYMERS syndrome and in genuine mania (references [71,72,73,74,75]), a close relationship between suffering and the dysregulation of rhythms emerges. Although at the moment this element has a merely heuristic value, given the relevance in terms of public health, this study suggests that the link between adaptive hyperactivity and the good regulation of biorhythms and social rhythms should be verified through the conduction of ad hoc studies better equipped in terms of the number of samples examined and the use of robust methodologies.

Given the preliminary nature of this study, other limits are, first of all, its inability to provide a truly representative sample of the general population of the blue zone and an adequate control sample. Furthermore, comparison of the frequency mean scores for PHQ9, SF-12, and MDQ positivity in our sample with the data of the general population could only be conducted for the group of elderly people over 64 years of age because frequency among old older people was not reported in the results of the Italian study. This could “dilute” the frequency of our sample of old older people in the entire sample of elderly people.

## 5. Conclusions

This study provides insight into the adaptive hyperactivity observed in older adults from Sardinia’s blue zone, emphasizing that such hyperactivity does not correlate with dysregulated social rhythms, depressive symptoms, or a diminished quality of life. These findings suggest that the hyperactivity prevalent in this population may be an adaptive trait contributing to their longevity and resilience. However, this study’s cross-sectional design limits causal inferences, and the relatively small sample size from a single geographical location may affect its generalizability. Additionally, comparisons with national data are constrained by variations in measurement tools and population characteristics. Despite these limitations, this study’s unique focus on a population with notable longevity offers valuable insights into the relationship between lifestyle factors and mental health. The use of well-validated instruments for mood assessment and the inclusion of biochemical markers further strengthen the findings. In summary, the results indicate that adaptive hyperactivity among elderly individuals in this blue zone population may be considered beneficial rather than pathological, warranting future research with larger and more diverse samples to explore the underlying mechanisms and potential protective role of this trait against mood disorders.

## Figures and Tables

**Table 1:** Study sample.

Item	Adults (*n* = 33)	Elders (*n* = 41)	Old Elders (Blue Zone) (*n* = 21)	Statistics
Sex (Female) (N)	13 (39.39%)	17 (41.46%)	5 (23.80%)	Chi-square = 0.032 *p* = 0.857
Age (years)	43.41 ± 18.77	72.59 ± 8.73	84.33 ± 3.94	ANOVA 1;72 df (Bonferroni) F = 78.262 *p* < 0.001

**Table 2:** Comparison with normative data of the sample variables available in the national (MDQ) sample.

Item	Old (*n* = 62)	Adults (*n* = 33)	Statistics of Old vs. Adults
SF12 Sample	33.41 ± 6.06 (62)	36.14 ± 5.28 (33)	One-way ANOVA (Bonferroni) 1;93 df; F = 4.766; *p* = 0.032
SF12 Italian Community	34.32 ± 7.20 (379)	38.61 ± 6.33 (1623)	One-way ANOVA (Bonferroni) 1;2000 df; F = 144.7; *p* < 0.0001
Statistics Sample vs. Community	ANOVA 1;439 df (Bonferroni) F = 0.887 *p* = 0.347	ANOVA 1;653 df (Bonferroni) F = 4.954 *p* = 0.026	

**Table 3:** Comparison with normative data of the sample variables available in the national (SF-12) sample.

Item	Old (*n* = 62)	Adults (*n* = 33)	Statistics of Old vs. Adults
MDQ+	14/62 (22.58%)	2/33 (6.06%)	Chi-square = 4.197 *p* = 0.041 OR = 4.52 CL 95% (1.0–21.3)
MDQ Italian Community	6/685 (0.87%)	97/2713 (3.57%)	Chi-square = 13.559 *p* < 0.0001; OR = 0.24; CL 95% (0.1–0.5)
Statistics Sample vs. Community	Chi-square = 155.83 *p* < 0.0001	Chi-square = 0.579 *p* = 0.447	

**Table 4:** Comparison with normative data of the sample variables available in the regional (PHQ9) sample.

Item	Old (*n* = 62)	Adults (*n* = 33)	Statistics of Old vs. Adults
PHQ9	4.01 ± 3.50	3.49 ± 2.89	One-way ANOVA (Bonferroni) 1;93 df; F = 0.601, *p* = 0.440
PHQ9 Italian Community	3.12 ± 3.53 (190)	2.85 ± 3.07 (530)	ANOVA 1;718 df (Bonferroni) F = 0.997; *p* = 0.318
Statistics Sample vs. Community	ANOVA 1;250 df (Bonferroni) F = 2.984; *p* = 0.088	ANOVA 1;261 df (Bonferroni) F = 1.350; *p* = 0.244	

**Table 5:** Comparison of oral VSC biomarkers and/or impairment and mood suffering within sub-groups of the study sample.

Item	Adults *n* = 33 (Pivot)	Young Old (>64; <80) *n* = 41	ANOVA 1;72 df (Bonferroni)	Old Old ≥80 *n* = 21	ANOVA 1;52 df (Bonferroni)
CH_3_SH 24 (ng/10 mL)	14.98 ± 15.33	12.52 ± 9.67	F = 0.707 *p* = 0.404	12.7 ± 14.57	F = 0.480 *p* = 0.491
(CH_3_)_2_S (ng/10 mL)	15.61 ± 23.64	24.13 ± 31.26	F = 1.677 *p* = 0.199	18.95 ± 25.52	F = 0.241 *p* = 0.626
BSRS	17.40 ± 6.09	20.64 ± 7.02	F = 4.376 *p* = 0.040	17.00 ± 5.08	F = 0.063 *p* = 0.803
PHQ9	3.49 ± 2.89	3.99 ± 4.02	F = 0.360 *p* = 0.550	4.05 ± 2.10	F = 0.589 *p* = 0.446
MDQ+	2 (6.45%)	8 (19.51%)	Fisher *p* = 0.088	6 (28.57%)	Fisher, *p* = 0.031
SF-12<31	4 (12.1%)	13 (31.70%)	Fisher *p* = 0.041	5 (23.80%)	Fisher *p* = 0.225

## Data Availability

The data presented in this study are available upon request from the corresponding author. The data are not publicly available due to privacy and ethical issues.
